# Anvisa authorization for electronic prescriptions of controlled substances: implications for equity in access to pain management in Brazil

**DOI:** 10.1590/0102-311XEN266325

**Published:** 2026-05-01

**Authors:** Guilherme Antonio Moreira de Barros, Claudia Marquez Simões

**Affiliations:** 1 Universidade Estadual Paulista, Botucatu, Brasil.; 2 Instituto do Câncer do Estado de São Paulo, São Paulo, Brasil.

## Introduction

### Equity in access to pain management

In Brazil, healthcare professionals - including physicians, dentists, and veterinarians - are required to use a specific type of prescription form (Type “A” pads, commonly referred to as*Receituários Amarelos* [yellow forms]) to dispense potent opioids and other controlled substances the Brazilian Health Regulatory Agency (Anvisa, acronym in Portuguese) classify as A1, A2, and A3. Health professionals cannot print these prescription forms; they must formally request them from the state or municipal health surveillance authority following prior professional registration. Healthcare institutions such as clinics and hospitals that meet the required documentation criteria and possess an official institutional stamp may also request these prescription forms ^1^.

Beyond the extensive documentation required to request Type A prescription forms, geographic constraints also require consideration. Despite no official national data available on the distribution of state or municipal health surveillance authorities across different municipalities, this requirement may represent a significant barrier in smaller towns. Brazil’s continental dimensions encompass highly isolated and remote communities. Many small towns in remote regions, such as the Amazon region, likely lack legally constituted health authorities who enjoy the authorization to dispense these prescription forms. Consequently, patients in these areas who require opioid analgesics for adequate pain management may face substantial obstacles in accessing appropriate treatment.

### Opioids as essential analgesics

The broad term “opioid” encompasses natural or synthetic substances whose pharmacological effect is mediated via an interaction with opioid receptors. Among these, morphine is the most widely recognized, and is considered as the gold standard analgesic within this class ^2^. These substances have significantly affected human behavior since antiquity, having historically served as sedatives and analgesics in therapeutic and non-medical contexts ^3^.

Although opioids remain essential analgesics in the clinical management of various pain conditions, their misuse can lead to significant medical and social consequences. The recreational opioid crisis affecting the population of several developed countries and its repercussions have received considerable attention in both the media and the scientific literature. These developments have contributed to a global rise in opiophobia (an irrational fear of prescribing or using opioids for pain management) ^4^.

Nevertheless, opioids remain among the most effective analgesics to treat moderate and severe pain despite ongoing controversies related to their adverse effects and potential for addiction ^4^ ^,^ ^5^. Furthermore, physicians often mention bureaucratic processes, limited pharmacological knowledge, and the regulatory frameworks governing opioid prescriptions as restrictive factors that hinder the appropriate use of these analgesics in certain regions of the world ^4^ ^,^ ^6^.

Nevertheless, a comprehensive population-based study in a municipality of approximately 150,000 inhabitants (Botucatu, São Paulo State, Brazil) on analgesic self-medication habits corroborates with this assertion. The highest pain intensity reported within the last 24 hours by the 190 subjects with chronic pain in that study yielded a 6.23 ± 2.81 mean score (on a 0-10 scale), a level that would justify incorporating opioids into their therapeutic protocol. However, only 2.6% of these subjects were on use weak opioids (mainly codeine). None received strong opioids ^7^. In another population-based study conducted in Michigan (United States) ^8^, 1.9% and 0.7% of respondents had used weak and strong opioids in the preceding two weeks, respectively. Notably, this study population had no chronic pain unlike the Brazilian survey referenced above.

## Development

In response to these challenges and following requests from the Brazilian medical community Anvisa implemented the Brazilian National Prescription Control System (SNCR, acronym in Portuguese) in 2024 - a digital platform designed to enhance safety in the issuance and use of prescriptions for medications subject to special control. Operational since May 2024 under *Resolution RDC n. 873/2024* ^9^, this system introduced electronic prescriptions (“e-prescriptions”), which have significantly transformed prescribing practices in Brazil.

This initiative represents a significant advancement in regulatory oversight and public health policy as it strengthens traceability, reduces the risk of diversion and misuse of controlled substances, and promotes greater standardization and transparency in prescription practices across Brazil. Up to the end of 2025, electronic prescriptions only included medications under the “prescription with retention” regimen, which requires pharmacies to retain simple prescription forms. The substances included in the e-prescription system comprised weak opioids, such as codeine and tramadol, anticonvulsants, and antidepressants. However, this scenario has recently changed ^9^.

In December 2025, Anvisa approved the inclusion of medications subject to stricter control to the SNCR, encompassing benzodiazepines and potent opioids, broadening prescriptions to A (yellow) and B (blue) forms. From this point forward, prescriptions for special-control medications and those requiring copy retention - such as antimicrobials and GLP-1 receptor agonist pre-filled injection pens - will only be valid when issued via electronic prescribing services within the SNCR that require the mandatory registration of each prescription within the system ^10^.

Another recent change is that only platforms that integrated within the SNCR are to generate electronic prescription forms. Paper prescriptions will remain in use, and no plans currently exist for their elimination. However, digital prescriptions will only be considered valid if processed by the SNCR ^10^. Although this measure may seem to increase the risk of misuse of certain medications (including strong opioids), its implications require evaluations from multiple perspectives. A recent scoping review on the impact of the adoption of e-prescriptions on opioid-related practices reported mixed findings ^11^.

### Impacts to the access and safety of opioids in Brazil

Approximately half of the studies included in the scoping review above - most of which examined experiences in the United States - found changes in prescribing rates, with some demonstrating reductions over time. Conversely, other studies observed an increase in opioid prescriptions following the implementation of computerized provider order entry, particularly in ambulatory care settings. Additionally, few studies reported reductions in the quantities of prescribed opioids, suggesting variability in outcomes across contexts and health systems. Furthermore, evidence indicates that e-prescribing systems have contributed to reducing preventable adverse drug events, including opioid-related oversedation, highlighting potential safety benefits associated with digital prescribing platforms ^11^.

Note that, a 2020 survey conducted with a representative sample of physicians (n = 389, 43.7% anesthesiologists) via an online questionnaire in Brazil identified the main barriers to prescribing strong opioids for analgesia. The most frequently reported difficulty referred to fear of inducing addiction (52.19% of respondents), followed by bureaucratic restrictions related to obtaining the specific prescription forms (50.13%) ^12^.

According to that study, physicians perceived that the primary factors that could facilitate opioid prescribing included improved knowledge of opioid pharmacology (73.01%), better understanding of the regulations governing their prescription (67.1%), and reduced bureaucratic requirements for obtaining the appropriate prescription forms (51.16%) ^12^. For these reasons, implementing e-prescriptions is expected to facilitate access to the required prescription forms, thereby enabling patients to obtain these analgesics regardless of their location.

As previously cited, electronic prescribing for controlled substances enhances the safety and traceability of opioid prescriptions by ensuring their electronical transmission to pharmacies, which reduces the risk of fraud and misuse commonly associated with paper prescriptions and improves patient safety and adherence ^11^ ^,^ ^13^.

Brazil still faces significant challenges in achieving universal access to pain management. However, gradual progress in the right direction has occurred. Its e-prescription system represents a major advancement. E-prescribing offers prescribers a safe and reliable mechanism to deliver pain relief to patients with legitimate clinical needs, reinforcing confidence in prescribing practices ^11^. The SNCR also provides prescribers with information regarding whether and when patients have collected their prescribed opioids from the pharmacy. Additionally, the system is expected to enable verification of potential “doctor shopping”, a practice in which patients seek multiple physicians to obtain a larger number of prescriptions for controlled substances ^14^.

However, medical education on the proper use of opioids is also essential. In the United States, opioid prescribing is contingent upon holding a valid registration with its Drug Enforcement Administration. Since 2023, clinicians applying for new or renewed registrations must also attest to the completion of at least eight hours of training on the treatment and comprehensive care of patients with opioid use disorder or other substance use disorders ^15^. This requirement reflects an effort to balance access to essential analgesic therapies with responsible prescribing and patient safety.

In Brazil, until a few years ago, medical schools often ignored pain management, a topic that still receives only superficial coverage in most curricula. Thus, supplemental and continuous education from medical societies plays a crucial role. A noteworthy example is the online course *Boas Práticas na Prescrição de Opioides* [Best Practices in Opioid Prescribing], an initiative of the São Paulo State Society of Anesthesiology, inspired by the United States initiative ^16^.

Nevertheless, the improved access to opioid e-prescriptions raises a justified concern regarding the chances of an opioid crisis in Brazil. The “debureaucratization” of access to prescription forms could excessively facilitate the availability of these analgesics. The previously existing bureaucratic restrictions on opioid use in Brazil, particularly for the more potent agents, may have functioned as a barrier against the emergence of an undesirable crisis of opioid overdoses due to recreational use - such as that in the United States. However, despite the absence of in-depth studies on the subject, we remain far from experiencing a scenario comparable to that observed in the United States ^4^ ^,^ ^17^ ^,^ ^18^.

Several probable reasons beyond the current prescribing bureaucracy may explain the absence of a recreational opioid crisis in Brazil, including their low availability and high cost for recreational use. Epidemiological data has indicated that the use of heroin is rare in Brazil and that non-medical use of prescribed opioids is significantly lower than in countries such as the United States, which faces a severe overdose crisis ^17^ ^,^ ^18^. The low prevalence of opioid use may also be related to patients’ lower acceptance of these analgesics (given the stigma stemming from their association with cancer and end-of-life situations) ^18^.

## Conclusions

Will the implementation of the e-prescribing system help bridge the substantial Brazilian gap in ensuring equitable access to pain management for those in need? Possibly yes. Such measures could also mitigate the risk of a recreational opioid crisis in this context. Conversely, may they expose the Brazilian population to a higher risk of the recreational use of these compounds? 

The coming years will be critical in answering these questions. For now, this initiative and its medical education improvement clearly represent a meaningful step forward - and perhaps a genuine ray of hope for patients living with pain.
